# A Comparison of Online Medical Crowdfunding in Canada, the UK, and the US

**DOI:** 10.1001/jamanetworkopen.2020.21684

**Published:** 2020-10-26

**Authors:** Sameh N. Saleh, Ezimamaka Ajufo, Christoph U. Lehmann, Richard J. Medford

**Affiliations:** 1 Department of Internal Medicine, The University of Texas Southwestern Medical Center, Dallas; 2 Clinical Informatics Center, The University of Texas Southwestern Medical Center, Dallas; 3 Departments of Pediatrics, Bioinformatics, Population & Data Sciences, The University of Texas Southwestern Medical Center, Dallas

## Abstract

**Importance:**

Despite major differences in their health care systems, medical crowdfunding is increasingly used to finance personal health care costs in Canada, the UK, and the US. However, little is known about the campaigns designed to raise monetary donations for medical expenses, the individuals who turn to crowdfunding, and their fundraising intent.

**Objective:**

To examine the demographic characteristics of medical crowdfunding beneficiaries, campaign characteristics, and their association with funding success in Canada, the UK, and the US.

**Design, Setting, and Participants:**

This cross-sectional study extracted and manually reviewed data from GoFundMe campaigns discoverable between February 2018 and March 2019. All available campaigns on each country domain’s GoFundMe medical discovery webpage that benefitted a unique patient(s) were included from Canada, the UK, and the US. Data analysis was performed from March to December 2019.

**Exposures:**

Campaign and beneficiary characteristics.

**Main Outcomes and Measures:**

Log-transformed amount raised in US dollars.

**Results:**

This study examined 3396 campaigns including 1091 in Canada, 1082 in the UK, and 1223 in the US. Campaigns in the US (median [IQR], $38 204 [$31 200 to $52 123]) raised more funds than campaigns in Canada ($12 662 [$9377 to $19 251]) and the UK ($6285 [$4028 to $12 348]). In the overall cohort per campaign, Black individuals raised 11.5% less (95% CI, −19.0% to −3.2%; *P* = .006) than non-Black individuals, and male individuals raised 5.9% more (95% CI, 2.2% to 9.7%; *P* = .002) than female individuals. Female (39.4% of campaigns vs 50.8% of US population; difference, 11.3%; 95% CI, 8.6% to 14.1%; *P* < .001) and Black (5.3% of campaigns vs 13.4% of US population; difference, 8.1%; 95% CI, 6.8% to 9.3%; *P* < .001) beneficiaries were underrepresented among US campaigns. Campaigns primarily for routine treatment expenses were approximately 3 times more common in the US (77.9% [272 of 349 campaigns]) than in Canada (21.9% [55 of 251 campaigns]; difference, 56.0%; 95% CI, 49.3-62.7%; P < .001) or the UK (26.6% [127 of 478 campaigns]; difference, 51.4%; 95% CI, 45.5%-57.3%; P < .001). However, campaigns for routine care were less successful overall. Approved, inaccessible care and experimental care raised 35.7% (95% CI, 25.6% to 46.7%; *P* < .001) and 20.9% (95% CI, 13.3% to 29.1%; *P* < .001), respectively, more per campaign than routine care. Campaigns primarily for alternative treatment expenses (16.1% [174 of 1079 campaigns]) were nearly 4-fold more common for cancer (23.5% [144 of 614 campaigns]) vs noncancer (6.5% [30 of 465 campaigns]) diagnoses.

**Conclusions and Relevance:**

Important differences were observed in the reasons individuals turn to medical crowdfunding in the 3 countries examined that suggest racial and gender disparities in fundraising success. More work is needed to understand the underpinnings of these findings and their implications on health care provision in the countries examined.

## Key Points

**Question:**

Why do individuals from Canada, the UK, and the US turn to medical crowdfunding and what factors are associated with funding success?

**Findings:**

In this cross-sectional study of 3396 crowdfunding campaigns designed to raise monetary donations for medical expenses, female gender, Black race, and routine care were associated with a strong fundraising disadvantage. For campaigns primarily funding treatment, routine care was overwhelmingly represented in the US (77.9%), in contrast to Canada (21.9%) and the UK (26.6%), and crowdfunding primarily funding alternative therapies (16.0%) was more common for cancer (23.5%) vs noncancer (6.5%) diagnoses.

**Meaning:**

These findings suggest that there are important differences in the reasons for medical crowdfunding across the 3 countries included in this analysis and that there are racial and gender disparities in crowdfunding success.

## Teaser

This cross-sectional study examines medical crowdfunding campaigns in Canada, the UK, and the US and evaluates whether campaign types and demographic characteristics of the beneficiaries are associated with the amounts of money raised.

## Introduction

Crowdfunding, the online solicitation of public donations, has become an important form of financing to pay for accumulated personal health care debts. Approximately one-third of all crowdfunding campaigns seek public monetary donations intended to pay for health care-related costs.^1,2,3^ The growing importance of medical crowdfunding (MCF) is reflected by trends on GoFundMe, the largest social crowdfunding platform in the world.^3,4^ In 2011, medical causes raised $1.6 million on GoFundMe; in 2014, the amount had increased almost a hundredfold to $150 million and in 2016, more than $650 million.^1,3^

The growing reliance of health care consumers from the US on MCF has been attributed to increasing health care costs and the lack of a publicly funded health care system.^1,5,6^ However, the popularity of MCF in developed countries with universal health care such as Canada and the UK^7,8,9,10^ cannot be similarly explained. To date, MCF has financed a range of therapies, including experimental and alternative therapies.^11,12,13,14^ However, inequity, barriers to access, invasion of privacy, fraud, and dangerous, unproven therapies have been associated with MCF, but are poorly understood.^4,7,15,16,17,18,19^ Despite its growth and the concerns surrounding crowdfunding, there is a paucity of empirical research on MCF, including research on sociodemographic characteristics of beneficiaries and the diagnoses and treatments championed.

The objective of this study was to evaluate 3 important areas of MCF: (1) the purpose for crowdfunding in terms of diagnoses and therapies funded, (2) the characteristics of beneficiaries and campaigns, and (3) the factors associated with funding success. We selected GoFundMe as an ideal environment to study. As of 2018, the platform reportedly controlled 90% of the social crowdfunding market in the US and 80% of the global market.^20^ We studied consecutive campaigns from the crowdsourcing platform in Canada, the UK, and the US, the 3 countries with the largest markets on this platform.

## Methods

### Study Population

**Figure 1.**
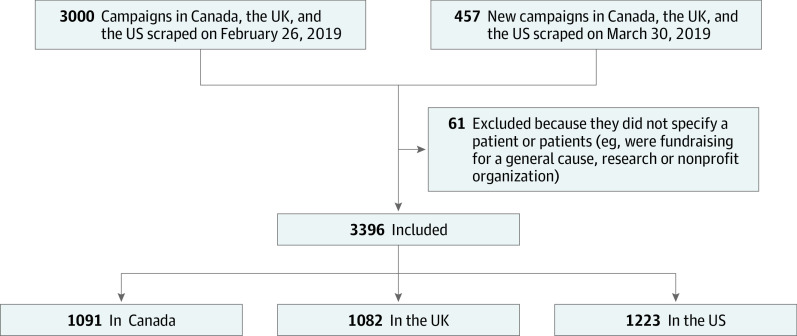
Study Flow Diagram

We conducted a cross-sectional analysis of campaigns launched between February 2018 and March 2019 from the GoFundMe domains in Canada, the UK, and the US. The crowdsourcing platform offers 21 cause categories for users to select; using a web scraping tool (Beautiful Soup^21^), we extracted campaigns from the medical category only. For each country’s domain, we accessed all 1000 available medical campaigns on the Discover page at 2 time points first in February 2019 and 30 days later in March 2019. Accounting for duplicates, our queries resulted in 1107 unique campaigns in Canada, 1117 in the UK, and 1232 in the US (Figure 1). We excluded 61 campaigns that were not fundraising for individuals (eg, raising funds for a general cause, research, or nonprofit organization). The University of Texas Southwestern Human Research Protection Program Policies, Procedures, and Guidance did not require institutional review board approval or informed patient consent as all data were publicly available. This study followed the relevant portions of the Strengthening the Reporting of Observational Studies in Epidemiology (STROBE) reporting guideline.^22^

### Study Variables

For each campaign, we extracted the quantitative data displayed on the campaign webpage, including the monetary goal, amount raised to date, number of donors, location, length of the fundraising campaign, Facebook shares, and GoFundMe hearts (the equivalent of webpage likes). Data were complete except for Facebook shares, with 2.6% of campaigns missing values, for which we imputed a value of zero. We converted all funds to US dollars based on currency exchange rates on the day we first accessed the data (Canadian dollar conversion rate of 0.76 on March 1, 2019, and British pound conversion rate of 1.32 on March 2, 2019).

Using the text and media on each initial campaign post, a 2-person manual review of the campaigns labeled data on demographic characteristics (including age, gender, and race), diagnosis, type of treatment (routine, experimental [ie, not yet approved], approved but inaccessible [ie, unavailable in the patient’s location], alternative [ie, treatments used in lieu of standard care], and unspecified), funding intent of the campaign (primarily for treatment costs or not), patient location (residing outside of the campaign country or not), and status (alive or deceased). The eAppendix in the Supplement describes in greater detail the variable definitions used. Each individual reviewed 55% of the data with 10% overlap between the reviewers. Interrater reliability (κ) greater than 0.8 indicates almost perfect agreement.^23^ Concordance analysis showed the κ value exceeded 0.77 for all but 1 category (eTable 1 in the Supplement). Age, diagnosis, gender, and race all had nearly perfect concordance (κ > 0.97). There was greater than 89.2% raw agreement in all categories, with most greater than 98% (eTable 1 in the Supplement). A blinded third reviewer adjudicated any discrepancies between the 2 reviewers.

### Statistical Analysis

We performed descriptive data analyses of all variables to evaluate trends and common characteristics of MCF in the 3 countries. Given the nongaussian populations, we used Kruskal-Wallis, χ^2^, and Fisher exact testing to detect statistical differences among the 3 groups. To test for representativeness, we compared the campaign demographics with respective national census data (2016 Canadian census,^24^ 2018 UK estimate,^25^ 2018 US estimate^26^) using a *z* test for proportions.

We performed multivariable linear regressions for the full cohort and by country using funds raised as the prespecified primary outcome variable as in prior studies.^11,12^ Given the right skewness of the outcome variable, we log-transformed it for the regression analysis. We constructed a pairwise Pearson correlation matrix and calculated the variance inflation factor among all variables and excluded repetitive variables with high collinearity from the analysis (eg, GoFundMe hearts [likes] and campaign narrative character count because of high correlation with the number of donors and narrative word count, respectively). To avoid multicollinearity in categorical variables, we dropped the normative or largest category to serve as the reference category (eg, adult for age, cancer for diagnosis, and routine care for the treatment type). We evaluated the models using the *R*^2^ coefficient. Because the outcome variable was log-transformed, we calculated percentage differences (and their 95% CIs) for the independent variables from the regression coefficients using the formula 100 × (*e^β^* − 1), where β is the corresponding regression coefficient and *e* is the natural logarithm (approximately 2.718). A categorical variable reflects the percentage difference in the dependent variable (ie, amount raised) as compared with the reference level. A continuous variable reflects the percentage difference in the dependent variable (ie, amount raised) with a 1-unit change in the independent variable.

The α level of significance was set a priori at .05, and all hypothesis testing was 2-sided. We did not adjust for multiple comparisons as this was an exploratory study and should be interpreted as hypothesis generating. Inferences may not be reproducible, and further dedicated studies are needed to confirm the results. Statistical analyses were performed using Python statistical software version 3.7.2 (Python Software Foundation) from March to December 2019.

## Results

### Beneficiary Demographic Characteristics and Diagnoses

Of the 3396 campaigns, 1091 originated in Canada, 1082 in the UK, and 1223 in the US. Table 1 presents the campaign characteristics, stratified by country. Most campaign beneficiaries were male (1767 beneficiaries [52.0%]), adult (2583 beneficiaries [76.1%]), and non-Black (3255 beneficiaries [95.8%]). Cancer (1850 campaigns [54.5%]) was the most common diagnosis represented in fundraisers, followed by neurologic (521 campaigns [15.3%]), other (353 campaigns [10.4%]), and trauma (332 campaigns [9.8%]) diagnoses.

**Table 1.** Baseline Characteristics Stratified by Country for Entire Cohort

Characteristic	Campaigns, No. (%)				*P* value^a^
	All (N = 3396)	Canada (n = 1091)	UK (n = 1082)	US (n = 1223)	
Age group					
Adult, >18 y	2583 (76.1)	803 (73.6)	813 (75.1)	967 (79.1)	.006
Minor, 2-18 y	604 (17.8)	213 (19.5)	212 (19.6)	179 (17.8)	.002
Infant, <2 y	139 (4.1)	60 (5.5)	34 (3.1)	45 (4.1)	.01
>1 Age group^b^	70 (2.1)	15 (1.4)	23 (2.1)	32 (2.6)	.11
Gender					
Male	1767 (52.0)	553 (50.7)	492 (45.5)	722 (59.0)	<.001
Female	1540 (45.3)	521 (47.8)	550 (50.8)	469 (38.4)	<.001
Transgender	14 (0.4)	0	14 (1.3)	0	<.001
>1 Gender^b^	75 (2.2)	17 (1.6)	26 (2.4)	32 (2.6)	.20
Race					
Black	141 (4.2)	21 (1.9)	55 (5.1)	65 (5.3)	<.001
Not Black	3255 (95.8)	1070 (98.1)	1026 (94.9)	1158 (94.7)	
Beneficiary outside country^c^	140 (4.1)	30 (2.7)	105 (9.7)	6 (0.5)	<.001
Diagnosis					
Acute illness	124 (3.7)	34 (3.1)	31 (2.9)	59 (4.8)	.02
Cancer	1850 (54.5)	659 (60.4)	532 (49.2)	659 (53.9)	<.001
Cardiac	108 (3.2)	19 (1.7)	33 (3.0)	56 (4.6)	<.001
Neurologic	521 (15.3)	158 (14.5)	218 (20.1)	145 (11.9)	<.001
Transplant	108 (3.2)	45 (4.1)	17 (1.6)	46 (3.8)	.001
Trauma	332 (9.8)	90 (8.2)	70 (6.5)	172 (14.1)	<.001
Other	353 (10.4)	86 (7.9)	181 (16.7)	86 (7.0)	<.001
Fundraising					
Goal, median (IQR), US $^d^	30 000 (11 400-57 000)	19 000 (10 260-38 000)	13 200 (5534-33 396)	50 000 (35 000-100 000)	<.001
Raised, median (IQR), US $^d^	18 505 (8570-36 052)	12 662 (9377-19 251)	6285 (4028-12 348)	38 204 (31 200-52 123)	<.001
Met goal	1133 (33.4)	415 (38.0)	333 (30.7)	385 (31.4)	<.001
Donors, median (IQR), No.	190 (106-332)	153 (104-232)	110 (68-194)	321 (222-498)	<.001
Primarily funding treatment^e^	1079 (31.8)	251 (23.0)	478 (44.2)	349 (28.5)	<.001
Campaign information, median (IQR)					
Facebook shares, No.	632 (306-1100)	565 (316-926)	386 (174-808)	1000 (547-1700)	<.001
GoFundMe hearts, No.	187 (104-331)	155 (103-232)	109 (67-191)	322 (222-506)	<.001
Fundraising length, mo	4 (1-6)	5 (2-8)	5 (1-8)	3 (1-4)	<.001
Narrative					
Words	317 (193-512)	321 (201-509)	343 (193-566)	301 (188-478)	.003
Characters	1844 (1118-2.978)	1884 (1172-2983)	1942 (1090-3256)	1762 (1091-2819)	.02

Abbreviation: IQR, interquartile range.

^a^ Given the nongaussian populations, we used Kruskal-Wallis, χ^2^, and Fischer exact testing to detect statistical differences among the 3 groups.

^b^ More than 1 beneficiary was included in the same campaign so no singular age and/or gender could be identified.

^c^ Beneficiary lives in a different country than the campaigning country (eg, Canadian campaign raising funds for family member in the Philippines).

^d^ All monetary values were converted to US dollars based on currency exchange rates at the time the data was accessed (March 1, 2019, for Canadian dollar; and March 2, 2019, for British pound).

^e^ Campaign clearly identifies that its primary funding goal is for medical treatment. See eAppendix in the Supplement for details.

Table 2 compares the campaign demographic characteristics in each country to its national census. The US had the highest proportion (59.0%) of male beneficiaries (Canada vs US and UK vs US, both *P* < .001). Female individuals comprise 50.8% of the US population, but were beneficiaries in only 39.4% of US campaigns (difference, 11.3%; 95% CI, 8.6%-14.1%; *P* < .001). In the US, adults were overrepresented compared with the census (81.2% vs 77.6%; difference, 3.6%; 95% CI, 1.4%-5.8%; *P* = .003). The US had more Black beneficiaries than Canada and a similar proportion to the UK. However, compared with national census representations, Black beneficiaries were most underrepresented in the US (5.3% vs 13.4%; difference, 8.1%; 95% CI, 6.8%-9.3%; *P* < .001). Black Canadians were also underrepresented (1.9% vs 3.5%; difference, 1.6%; 95% CI, 0.7%-2.4%; *P* = .004), and Blacks in the UK were overrepresented in campaigns (5.1% vs 3.3%; difference, 1.8%; 95% CI, 0.7%-3.3%; *P* < .001).

**Table 2.** Comparison of the Proportion of Campaign Representation to the Proportion of Population According to National Census Data (2016 Canada, 2018 UK, 2018 US)^a^

Characteristics	Canada			UK			US		
	Campaigns, No. (%)	Census, %	*P* value^b^	Campaigns, No. (%)	Census, %	*P* value^b^	Campaigns, No. (%)	Census, %	*P* value^b^
Age group									
Adult, >18 y	803 (74.8)	80.0	<.001	813 (76.8)	78.8	.10	967 (81.2)	77.6	.003
Minor, 2-18 y	213 (19.8)	17.9	.11	212 (20.0)	18.9	.34	179 (15.0)	20.1	<.001
Infant, <2 y	60 (5.6)	2.1	<.001	34 (3.2)	2.3	.03	45 (3.8)	2.4	.001
Gender									
Male	553 (51.5)	49.1	.12	492 (47.2)	49.3	.18	722 (60.6)	49.2	<.001
Female	521 (48.5)	50.9		550 (52.8)	50.7		469 (39.4)	50.8	
Race									
Black	21 (1.9)	3.5	.005	55 (5.1)	3.1	<.001	65 (5.3)	13.4	<.001
Not Black	1070 (98.1)	96.5		1026 (94.9)	96.9		1158 (94.7)	86.6	

^a^ Campaigns with more than one beneficiary were excluded.

^b^ The *P* value is determined by using the *z* test to compare the proportion of campaign representation with the proportion of the national census in each country.

### Fundraising Characteristics

Campaigns in this study collectively raised $92.9 million, accounting for 51.9% of the total funds sought. US campaigns (median [IQR], $38 204 [$31 200 to $52 123]) raised more funds than campaigns in Canada ($12 662 [$9377 to $19 251]) and the UK ($6285 [$4028 to $12 348]). Only 33.3% of all campaigns had met their goal at data extraction. Funds raised ranged from $2772 to $343 762 per campaign. US campaigns set higher fundraising goals than those in Canada and the UK (Table 1). Accounting for $57.8 million (62.2% of the total funds raised), US campaigns raised approximately 3 times the funds of Canadian campaigns and 6 times the funds of UK campaigns on average, despite being 2 months shorter in duration. US campaigns had significantly more donors and Facebook shares compared with campaigns in Canada and the UK.

### Treatment Type

**Figure 2.**
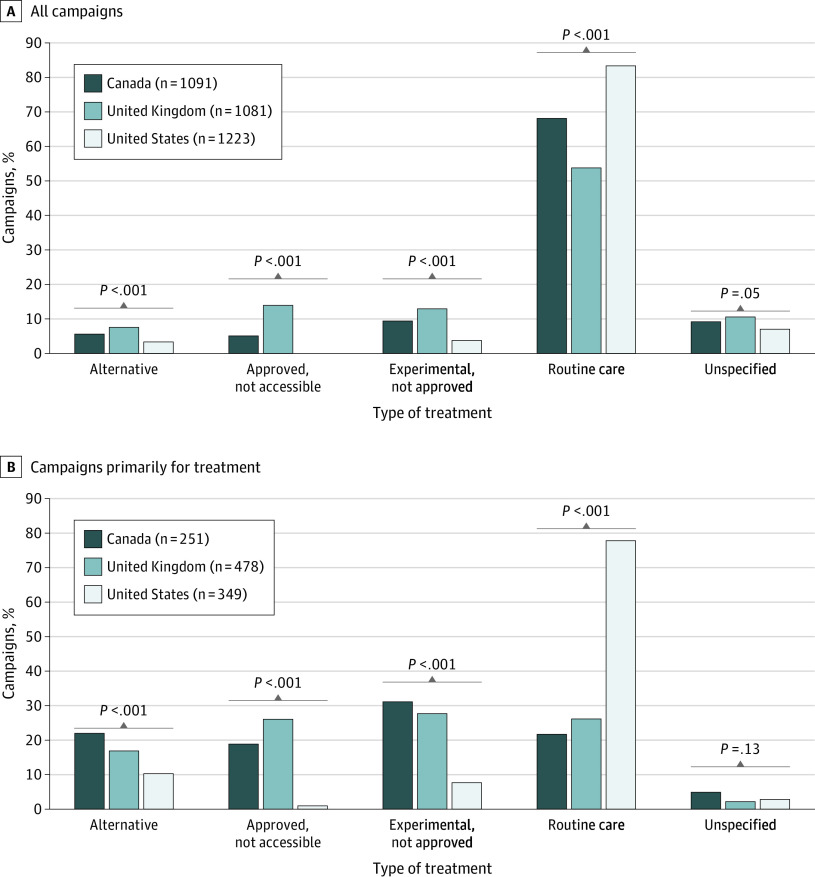
Primary Type of Treatment by Country Graphs show type of treatment for all campaigns (A) and for campaigns primarily for treatment costs (B). χ^2^ testing was used to derive *P* values comparing each type of treatment in the 3 countries.

Figure 2 summarizes the treatment types for which campaigns raised funding, stratified by country. Although routine care was the most common treatment type, accounting for 69.4% of all campaigns, 83.9% of US campaigns focused on routine care compared with 68.5% of Canadian campaigns (difference, 15.4%; 95% CI, 12.0%-18.9%; *P* < .001) and 54.0% of UK campaigns (difference, 29.9%; 95% CI, 26.3%-33.5%; *P* < .001) (Figure 2A). Experimental, approved but inaccessible, and alternative care were all more common among Canadian and UK campaigns compared with US campaigns. The UK had substantially more approved but inaccessible care than Canada (14.1% vs 6.0%; difference, 8.1%; 95% CI, 5.6%-10.6%; *P* < .001) or the US (14.1% vs 0.3%; difference, 13.8%; 95% CI, 11.6%-15.8%; *P* < .001). Approximately one-third of all campaigns (1078 campaigns) were fundraising primarily for treatment expenses. Of these, campaigns funding routine care were approximately 3 times more common in the US (77.9% [272 of 349 campaigns]) than in Canada (21.9% [55 of 251 campaigns]; difference, 56.0%; 95% CI, 49.3-62.7%; *P* < .001) or the UK (26.6% [127 of 478 campaigns]; difference, 51.4%; 95% CI, 45.5%-57.3%; *P* < .001) (Figure 2B). Campaigns primarily for treatment expenses in Canada and the UK were similarly distributed among routine, alternative, experimental, and unavailable therapies. Combined, fundraising for experimental and approved but inaccessible therapies was the purpose of approximately 50% of campaigns both in Canada and in the UK. Campaigns primarily for alternative treatment expenses (16.1% [174 of 1079 campaigns]) were nearly 4-fold more common for cancer (23.5% [144 of 614 campaigns]) vs noncancer (6.5% [30 of 465 campaigns]) diagnoses (eTable 2 in the Supplement).

### Associations With Campaign Success

Findings from multivariable regression analysis of the log-transformed funds raised per campaign are presented in Table 3. Campaign country was most strongly associated with the amount raised in the full cohort. Campaigns from Canada and the UK yielded 59.1% (95% CI, −61.0% to −57.1%; *P* < .001) and 78.4% (95% CI, −79.4% to −77.3%; *P* < .001) less funding than US campaigns, respectively. Number of donors and fundraising goals were strongly associated with funding success (Table 3). Facebook shares were weakly associated with funding success in the main regression model, but when donor numbers (moderately collinear with Facebook shares) were removed from the model, Facebook shares became strongly associated with funds raised (data not shown).

**Table 3.** Percentage Differences in Amount Raised for the Full Cohort and for Each Country

Characteristics	Differences, % (95% CI)^a^							
	All (*R*^2^ = 0.72)	*P* value	Canada (*R*^2^ = 0.41)	*P* value	UK (*R*^2^ = 0.52)	*P* value	US (*R*^2^ = 0.47)	*P* value
Demographic characteristics and location								
Gender								
Female	0 [Reference]	NA	0 [Reference]	NA	0 [Reference]	NA	0 [Reference]	NA
Male	5.9 (2.2 to 9.7)	.002	10.9 (5.4 to 16.7)	<.001	5.2 (−3.0 to 14.1)	.22	4.5 (1.0 to 8.2)	.01
Transgender	−26.7 (−44.2 to −3.7)	.03	NA	NA	−19.9 (−43.8 to 14.2)	.22	NA	NA
Age								
Adult	0 [Reference]	NA	0 [Reference]	NA	0 [Reference]	NA	0 [Reference]	NA
Minor	−5.4 (−9.7 to −0.9)	.02	−5.5 (−11.6 to 1.0)	.10	−6.3 (−15.2 to 3.6)	.20	−1.9 (−6.5 to 3.0)	.82
Infant	−12.5 (−20.0 to −4.4)	.003	−8.6 (−18.4 to 2.4)	.12	−21.1 (−37.1 to −0.8)	.04	−1.0 (−9.6 to 8.3)	.45
Black race	−11.5 (−19.0 to −3.2)	.006	−15.8 (−30.0 to 1.2)	.07	−10.3 (−25.8 to 8.4)	.26	−3.7 (−10.7 to 3.9)	.33
Country domain								
US	0 [Reference]	NA	0 [Reference]	NA	0 [Reference]	NA	0 [Reference]	NA
Canada	−59.1 (−61.0 to −57.1)	<.001	NA	NA	NA	NA	NA	NA
UK	−78.4 (−79.4 to −77.3)	<.001	NA	NA	NA	NA	NA	NA
Non-Medicaid expansion	NA	NA	NA	NA	NA	NA	1.4 (−2.4 to 5.4)	.48
Beneficiary outside country^b^	**−**0.2 (−9.3 to 9.7)	.96	−2.6 (−17.3 to 14.7)	.75	4.8 (−10.7 to 22.9)	.57	−8.5 (−28.2 to 16.5)	.47
Beneficiary deceased	−6.0 (−16.1 to 5.5)	.29	3.5 (−15.6 to 26.9)	.74	−13.9 (−34.0 to 12.3)	.27	−7.3 (−15.8 to 2.0)	.12
Diagnosis								
Cancer	0 [Reference]	NA	0 [Reference]	NA	0 [Reference]	NA	0 [Reference]	NA
Acute illness	−9.2 (−17.0 to −0.7)	.03	−14.4 (−25.7 to −1.4)	.03	−7.0 (−25.5 to 16.1)	.52	−7.5 (−14.4 to −0.1)	.05
Cardiac	−0.6 (−9.4 to 9.1)	.90	−5.1 (−21.6 to 14.9)	.59	−6.5 (−25.8 to 17.9)	.57	2.4 (−4.8 to 10.1)	.52
Neurologic	0.0 (−4.7 to 5.0)	.99	−4.3 (−11.2 to 3.0)	.24	6.5 (−3.6 to 17.7)	.22	5.2 (−0.1 to 10.7)	.05
Transplant	−12.2 (−19.9 to −3.8)	.005	−17.5 (−26.8 to −7.0)	.002	−5.2 (−28.8 to 26.2)	.72	−4.8 (−12.3 to 3.4)	.24
Trauma	−6.6 (−12.1 to −0.7)	.029	−0.1 (−9.2 to 9.9)	.98	−6.1 (−20.0 to 10.2)	.44	−1.7 (−6.7 to 3.5)	.50
Treatment type and details								
Routine care	0 [Reference]		0 [Reference]		0 [Reference]		0 [Reference]	
Alternative	6.5 (−0.7 to 14.3)	.08	7.2 (−3.4 to 18.9)	.19	7.9 (−7.4 to 25.8)	.33	0.9 (−6.3 to 8.6)	.81
Approved (not accessible)	35.7 (25.6 to 46.7)	<.001	25.8 (11.6 to 41.8)	<.001	24.4 (8.7 to 42.3)	.001	70.6 (27.0 to 129.0)	<.001
Experimental (not approved)	20.9 (13.3 to 29.1)	<.001	5.1 (−4.6 to 15.8)	.31	21.5 (5.1 to 40.4)	.008	11.4 (3.6 to 11.4)	.004
Campaign information and social media								
Goal, in $10 000 US^c^	0.7 (0.6 to 0.7)	<.001	2.8 (2.1 to 3.5)	<.001	1.5 (0.9 to 2.1)	<.001	0.4 (0.3 to 0.5)	<.001
Fundraising length, mo	1.2 (0.6 to 1.8)	<.001	2.5 (1.6 to 3.3)	<.001	2.8 (1.6 to 4.0)	<.001	3.2 (2.1 to 4.3)	<.001
Primarily funding treatment^d^	0.5 (−4.1 to 5.3)	.85	−1.3 (−9.0 to 7.0)	.75	1.1 (−10 to 13.7)	.85	−6.6 (−10.2 to −2.9)	.001
Narrative, in 100s of words	0.8 (0.3 to 1.3)	.003	0.9 (0.2 to 1.5)	.02	1.7 (0.6 to 2.9)	.003	0.1 (−0.4 to 0.6)	.79
Donors, in 100s	8.5 (7.9 to 9.2)	<.001	8.7 (7.3 to 10.2)	<.001	8.1 (6.3 to 10.0)	<.001	4.8 (4.3 to 5.3)	<.001
Facebook shares, in 100s	0.3 (0.1 to 0.5)	<.001	0.4 (−0.1 to 0.8)	.16	1.4 (0.8 to 2.0)	<.001	0.1 (−0.1 to 0.2)	.27

^a^ Values (and their 95% CI) are derived from a multivariable linear regression using an outcome variable of log-transformed amount raised using the formula 100 × (*e^β^* − 1), where β is the corresponding regression coefficient. For categorical variables, values reflect percentage difference in amount raised as compared with the reference level. For continuous variables, values reflect percentage difference in amount raised with a 1-unit change in the variable.

^b^ Beneficiary of the campaign lives in a different country than a campaigning country (eg, Canadian campaign raising funds for family member in the Philippines).

^c^ All monetary values were converted to US dollars based on currency exchange rates at the time the data was accessed. See Methods for details.

^d^ Campaign clearly identifies that its primary funding goal is for medical treatment. See eAppendix in the Supplement for details.

The gender and race of beneficiaries were also associated with funding success. Overall, Black beneficiaries raised 11.5% less per campaign (95% CI, −19.0% to −3.2%; *P* = .006) with concordant but nonsignificant trend in the country-specific analyses. Overall, male beneficiaries raised 5.9% more per campaign than their female counterparts (95% CI, 2.2% to 9.7%; *P* = .002); this trend was most pronounced in Canadian campaigns with male individuals raising 10.9% more than female individuals (*P* < .001). Campaigns for approved but inaccessible care and experimental care raised 35.7% (95% CI, 25.6% to 46.7%; *P* < .001) and 20.9% (95% CI, 13.3% to 29.1%; *P* < .001) more than campaigns for routine care, respectively. The association was strongest in the US where campaigns for approved but inaccessible care received 70.6% (95% CI, 27.0% to 129.0%; *P* < .001) more per campaign than those for routine care.

## Discussion

We examined 3396 medical campaigns from Canada, the UK, and the US, the 3 largest crowdfunding markets on GoFundMe, to characterize MCF beneficiaries, reasons for MCF, and factors associated with funding success. To our knowledge, this is the largest quantitative analysis of the MCF landscape to date. US campaigns set higher goals and raised more funds than campaigns from Canada or the UK. However, approximately two-thirds of campaigns in each country did not meet their funding goals. In the US, nearly 80% of campaigns primarily funding treatment were for routine care, whereas in Canada and the UK, funding for routine care was sought about as frequently as funding for alternative, approved but inaccessible, and experimental therapies. Campaigns for routine care were negatively associated with crowdfunding success, whereas campaigns for experimental and approved but inaccessible therapies were positively associated with funding success. Finally, we observed significant gender and racial inequities among beneficiaries.

### Beneficiary Demographic Characteristics and Inequities in Crowdfunding

Crowdfunding was initially heralded as a digital safety net or a mechanism for democratizing charity where anyone could benefit.^7^ However, anecdotal and empirical evidence suggests that crowdfunding may exacerbate socioeconomic inequities.^9,15,27^ In our study, Black and female individuals were underrepresented in US campaigns and Black individuals were underrepresented in Canadian campaigns. In another study of 637 randomly sampled US MCF campaigns from GoFundMe, non-White beneficiaries were also significantly underrepresented, constituting only 19% of the sample although this group represents 27% of the US population.^28^ Within our sample, female and Black beneficiaries raised 5.9% and 11.5% less than their male and non-Black counterparts, respectively. Similarly, in a US study of 850 campaigns for organ transplantation, female individuals raised 27% less than male individuals.^11^ In a Canadian study of 319 campaigns, being a visible ethnic minority was associated with raising 15% less in funds, before adjustment for technological competency (using quantity of campaign images, videos, updates, and perks) and 6% less after adjustment.^7^

Race and gender disparities reflect the pillars on which MCF is dependent: access to technology, literacy, social capital, and perception. Those with socioeconomic disadvantage are more likely to experience a digital divide that limits online participation because of a lack of access to information technology (eg, computer and internet). Writing, media, and health care literacies, which reflect socioeconomic privilege, enable an individual to augment his or her illness narrative, communicate deservingness, and thereby, generate campaign appeal and influence.^4^ Furthermore, broader, more affluent social communities and larger social media networks are well-recognized factors associated with crowdfunding success.^4,7,11^ Finally, conscious and unconscious systemic racial and gender biases likely obscure perception of worthiness in MCF campaigns.^29^ Our findings suggest that MCF facilitates the distribution of resources according to biases and preferences and thus, contribute to widening social inequities.^30,31^

### Reasons Individuals Turn to Medical Crowdfunding and Clues to Possible Gaps in Health Care Funding

If trends in MCF reflect health care costs and gaps in insurance coverage,^7,31^ our findings may point to possible country-specific shortcomings in health care funding. The trends seen in the US suggest that its greatest systemic funding failure may lie in the provision of routine health care. Despite a decrease in the uninsured rate after passage of The Affordable Care Act, as of 2018, 11.1% of adults younger than 65 years were uninsured^32^ and 29% were estimated to be underinsured^33^ (ie, out-of-pocket costs or deductibles comprising 5% to 10% of their income). For US campaigns across all diagnoses, and especially for common diagnoses such as acute illness and trauma, routine care was the most common reason for fundraising. Among campaigns primarily funding treatments, 77.9% were for routine care in the US compared with 21.9% and 26.6% of analogous campaigns in Canada and the UK, respectively. Interestingly, campaigns for routine care raised substantially less in the US, perhaps reflecting the saturation of the US MCF market with this type of campaign. US health care costs are the highest in the developed world, and health insurance deductibles have increased 8 times as much as wages since 2008.^34^ The disproportionate popularity of MCF in the US, combined with the predominance of campaigns for routine care, likely reflect high out-of-pocket costs associated with essential health care in the US.

The trends seen in Canada and the UK may reflect unique failures within their publicly funded health care systems. Routine care comprised 21.9% and 26.6% of campaigns primarily funding treatment in Canada and the UK, respectively, pointing to possible gaps despite universal health care. These gaps may be associated with rising out-of-pocket medication costs and insurance premiums in Canada and dissatisfaction with wait times for public care in the UK.^7,10,35^ Fundraising for experimental and approved but inaccessible therapies was the purpose of approximately 50% of campaigns in Canada and the UK, highlighting one of the perceived shortcomings of publicly funded health care—insufficient or delayed access to novel and experimental treatments.

For patients with cancer, financial hardship is common as novel expensive therapies become the standard of care and survival rates rise.^36,37,38^ Of the 9.5 million people diagnosed with cancer between 2000 and 2012 in the US, 42.4% had exhausted their life’s financial assets within 2 years.^39^ The enormous scope of unmet financial need in cancer care is reflected in the marked overrepresentation of cancer campaigns on crowdfunding platforms.^4,7,14^ In our cohort, cancer accounted for 54.6% across the 3 countries. Notably, raising funds for alternative therapies was far more common in cancer campaigns primarily funding treatment than for the other diagnoses examined. Patients may seek alternative therapies for cancer to complement or replace proven treatments either by choice or because they were not available or failed.^13^ The popularity of campaigns for alternative therapies for cancer potentially propagates unproven and potentially dangerous therapies yielding wasted resources, false hopes, delay of appropriate palliative care, and reduced survival.^13,16,18^

### Factors Associated With Crowdfunding Success

Beneficiary demographic characteristics, treatment type, the campaigning country, number of donors, fundraising goal, and campaign narrative length were important factors associated with funding success. Initial regression analysis did not demonstrate a fundraising association with Facebook shares, but further sensitivity analysis that excluded donor numbers from the model, showed an association between Facebook shares and funds raised, reflecting collinearity between Facebook shares and donor numbers. Previous crowdfunding research has shown that demographic characteristics,^7,11^ donor numbers,^40^ fundraising goal,^11,12^ campaign narrative length,^11,12,41^ and social media presence^4,7,41^ are associated with crowdfunding success. Setting a higher goal for medical campaigns may indirectly communicate depth of need to donors and promote the concept of deservingness. Campaign narrative length reflects the importance of the illness narrative to funding success. On the basis of previous research, we hypothesize that positive language in the narrative confers a fundraising advantage,^11,41^ whereas references to the systemic injustices that led to a given MCF campaign are infrequently observed.^42^

### Limitations

This study has several limitations. First, although our data set is larger than those in previous MCF research, it is only a small subset of popular MCF campaigns. Only campaigns visible on the Discover page of GoFundMe were included in our sample, which might have led to a bias toward more successful campaigns. We adopted this approach because it allowed us to filter out dummy or unverifiable campaigns that would have made our analysis unreliable. Further work is needed to explore trends in all campaigns and other factors affecting funding success. Generalizability of our findings is limited by the use of only 1 MCF platform. It is difficult to truly ascertain the representation of MCF platforms because of a paucity of historical data and inaccessibility of proprietary information in a commercial market. Second, the veracity of the online data cannot be ensured, which restricts interpretability, but also highlights the potentially important problems of misinformation, pseudoscience, and fraud in MCF. The lack of regulation and oversight raises questions about legal and medical responsibility. Third, there are inherent limitations in manual review, especially for demographic data using only media and textual context. However, in the absence of auditable information, we believe this method provides an adequate granular view of the campaign information and demographic characteristics in MCF. Near-perfect concordance between reviewers also shows the reliability of the coded data. Moreover, we believe our approach parallels the online crowdfunding experience of a potential donor who would rely on his or her perception of a beneficiary’s identity and attribute merit based on the illness narrative and media alone.

## Conclusions

We provide a foundational descriptive analysis of MCF and factors associated with success in Canada, the UK, and the US, and highlight important differences in MCF trends between publicly and privately funded health care systems. Our findings also suggest that there are racial and gender disparities in the use and success of MCF. MCF directly (through platforms that promote the victim narrative) and indirectly (by rewarding these narratives with funding success) promotes the myth that gaps in health care funding are due to misfortune and exceptionality, rather than systemic failures. As such, MCF may entrench the systemic failures that led to its need. Thus, although crowdfunding has the potential to provide short-term relief from medical financial burden for a subset of patients, it may carry wider-reaching paradoxical societal effect for those most socially disadvantaged. Further research is needed to understand the social, ethical, and economic implications of MCF within each health care setting and inform policy changes that promote equitable and accessible health care through this practice.

## Notes

Supplement. **eAppendix.** Manual Variable Definitions and Details **eTable 1.** Concordance Analysis for All Manual Review Variables **eTable 2.** Diagnosis Stratified by Type of Treatment Only for Campaigns Primarily Funding Treatment **eReferences.**
