# Supplementary material for: A Comparison of Online Medical Crowdfunding in Canada, the UK, and the US
Source: JAMA Netw Open. 2020 Oct 26;3(10):e2021684. doi: 10.1001/jamanetworkopen.2020.21684 (PMC7588935; doi:10.1001/jamanetworkopen.2020.21684)
Supplement: Supplement. — eAppendix. Manual Variable Definitions and Details eTable 1. Concordance Analysis for All Manual Review Variables eTable 2. Diagnosis Stratified by Type of Treatment Only for Campaigns Primarily Funding Treatment eReferences. [file jamanetwopen-e2021684-s001.pdf]

Supplemental Online Content

Saleh SN, Ajufo E, Lehmann CU, Medford RJ. A comparison of online medical crowdfunding in Canada, the UK, and the US. *JAMA Netw Open*. 2020;3(10):e2021684.
doi:10.1001/jamanetworkopen.2020.21684

eAppendix. Manual Variable Definitions and Details

eTable 1. Concordance Analysis for All Manual Review Variables

eTable 2. Diagnosis Stratified by Type of Treatment Only for Campaigns Primarily Funding Treatment

eReferences.

This supplemental material has been provided by the authors to give readers additional information about their work.

© 2020 Saleh SN et al. *JAMA Network Open.*

eAppendix. Manual Variable Definitions and Details

1) **Gender:** gender of the beneficiary
   - Labels and definitions:
     - Male
     - Female
     - Transgender
     - More than one beneficiary: More than one beneficiary was included in the same campaign so no singular gender could be identified.
   - Comments:
     - Identified based on visual context in media included and/or text description of the campaign.
2) **Age:** age of the beneficiary at the time of the start of the campaign
   - Labels and definitions:
     - Adult: > 18 years old
     - Minor: > 2 and ≥ 18 years old
     - Infant: ≤ 2 years old
     - More than one beneficiary: More than one beneficiary was included in the same campaign so no singular age could be identified.
   - Comments:
     - Identified based on visual context in media included and/or text description of the campaign.
3) **Race:** race of the beneficiary
   - Labels and definitions:
     - Black: Appears to have origins in any of the Black racial groups of Africa as defined by the US Census Bureau.
     - Non-black: Appears to be White, American Indian, Asian, and Native Hawaiian or Other Pacific Islander as defined by the US Census Bureau.
   - Comments:
     - Identified based on visual context in media included and/or text description of the campaign.
4) **Primary diagnosis:** diagnosis grouping for which the beneficiary is primarily fundraising.
   - Labels and definitions:
     - Acute illness: Results from an acute process, including but not limited to, infection including sepsis, complication of a procedure, and complication of pregnancy.
     - Cancer: Self-identified as "cancer" or is a known malignant process (e.g. leukemia, lymphoma, myeloma)
     - Cardiac: Directly involves or is a result of a heart condition. This includes, but is not limited to, abnormal heart rhythms, coronary artery disease, heart failure, heart valve disorders, congenital heart disease, heart surgeries, and cardiac arrest.
     - Neurologic: Directly involves the brain, spinal cord, or peripheral nerves. This includes, but is not limited to, amyotrophic lateral sclerosis, epilepsy or other seizure disorder, memory disorders, multiple sclerosis, Parkinson's disease, peripheral neuropathy, and strokes.
     - Transplant: Self-identified for organ transplantation or as a direct consequence post-transplantation including costs of immunosuppression, follow-up, and surveillance testing.
     - Trauma: Results directly from any physical trauma, including but not limited to, accidents, assault (physical or sexual), burns, and falls that lead to bodily harm.
     - Other: Diagnosis is not included in groups above or campaign does not identify a diagnosis.
   - Comments:
     - Identified based on the text description of the campaign.
     - Choice of diagnosis groupings included are based on previous common medical crowdfunding research topics[1–5].
5) **Secondary diagnosis:** second diagnosis grouping if the campaign either includes a separate second diagnosis group or the condition included also falls under a second diagnosis group.
   - Labels and definitions:
     - Same as 4) primary diagnosis.
   - Comments:
     - Left blank if no second diagnosis grouping is present.

© 2020 Saleh SN et al. *JAMA Network Open.*

6) **Primarily funding for treatment:** Campaign clearly identifies that its primary funding goal is for medical costs.

- Labels and definitions:
  - Binary [yes, no]
- Comments:
  - Identified based on either of the following:
    - Ask statement: the statement within the campaign text description that identifies what the patient is fundraising for.
    - An itemized allocation of funds
  - Must clearly specify the treatment and that either the sole or primary reason for the campaign is to directly fund medical costs. If multiple expenses are mentioned, medical costs must be explicitly stated to be the majority or primary expense.
  - Within scope:
    - Clinical evaluation/visits
    - Hospital bills
    - Diagnostic testing
    - Medical devices
    - Pharmacologic intervention
    - Surgery
  - Outside scope:
    - Nonmedical bills
    - Lost wages
    - Travel and lodging
    - Child care
    - Funeral costs
    - Charity donation
    - Rehabilitation (physical therapy, occupational therapy, speech therapy)
    - Home health
    - Clinical research
    - Cosmetic therapies

7) **Primary type of treatment:** treatment type that the beneficiary is primarily receiving at the time of and/or during the course of the campaign.

- Labels and definitions:
  - Alternative: Treatments that are used in place of standard of care or conventional medicine that are largely unproven, including but not limited to, acupuncture, energy therapies like reiki and magnet therapy, herbal preparations including special teas, homeopathy, megadose vitamins, and special diets.
  - Approved – Not Accessible: Treatments that have market approval for the diagnosis included and are considered standard of care, but are not available or accessible in the beneficiary's location or country (e.g. CAR T-cell therapy for refractory ALL or stereotactic radiosurgery for medulloblastoma).
  - Experimental – Not Approved: Treatments that do not have market approval for the diagnosis included and are not currently considered standard of care that are being used in an investigational capacity, including but not limited to, clinical trials, unproven surgical procedures, and off-label uses.
  - Routine: Treatments that have market approval and/or are considered standard of care for the diagnosis included.
  - Unspecified: Treatment is not specified, is unclear, or could not be ascertained since diagnosis is unclear.
- Comments:
  - Identified based on text description of campaign.
  - Standard of care determined based on clinical expertise of reviewers with market approval based on the Food and Drug Administration (FDA), Health Canada (HC), and European Union (EU).

8) **Second type of treatment:** treatment type if the patient is receiving a supplemental second type of treatment.

- Labels and definitions:
  - Same as 7) primary type of treatment.
- Comments:
  - Left blank if no second type of treatment is present.

© 2020 Saleh SN et al. *JAMA Network Open.*

9) **Experimental stem cell treatment:** Stem cell therapy is included in the treatment plan in an experimental or investigational capacity (e.g. for amyotrophic lateral sclerosis).
   - Labels and definitions:
     - Binary [yes, no]
   - Comments:
     - All that are positive will have "Experimental – Not Approved" as the type of treatment.
     - Does not include diagnoses approval for stem cell therapies, including but not limited to, leukemias, lymphomas, certain blood cell proliferative diseases, and certain inherited immune and metabolic disorders.
     - Included as a variable due to previous research exploring medical crowdfunding for experimental stem cell transplants[6].

10) **Beneficiary in another country:** Beneficiary of the campaign lives or is staying in a different country than the campaigning country (e.g. Canadian campaign raising funds for a family member in the Philippines).
   - Labels and definitions:
     - Binary [yes, no]
   - Comments:
     - Includes beneficiaries who are based in the campaigning country, but traveled to another country and incidentally required medical care.
     - Does not include beneficiaries that are centered in the campaigning country, but seek care in another country for alternative care or treatment being unavailable in the campaigning country.

11) **Campaign started posthumously:** the campaign was started after the beneficiary had already died.
   - Labels and definitions:
     - Binary [yes, no]
   - Comments:
     - Does not include campaigns where the beneficiary died while the campaign is ongoing, but after it was started.

© 2020 Saleh SN et al. *JAMA Network Open.*

eTable 1. Concordance Analysis for All Manual Review Variables. Table S1-A shows the Cohen's kappa interrater statistic and Table S1-B shows the raw percent match between reviewers.

**Table S1-A.**	**Cohen's Kappa (κ)**		
	**Canada (n = 111)**	**United Kingdom (n = 111)**	**United States (n = 124)**
**Gender**	1.00	1.00	1.00
**Age**	0.98	0.97	0.98
**Race**	1.00	1.00	1.00
**Diagnosis**	100	1.00	0.98
**Type of treatment**	0.84	0.87	0.77
**Experimental stem cell**	0.66	0.93	1.00
**Primarily funding treatment**	0.87	0.89	0.80
**Beneficiary outside country**	1.00	1.00	1.00
**Beneficiary deceased**	1.00	1.00	1.00
**Table S1-B.**	**Raw Match (%)**		
	**Canada (n = 111)**	**United Kingdom (n = 111)**	**United States (n = 124)**
**Gender**	100	100	100
**Age**	99.1	99.1	99.2
**Race**	100	100	100
**Diagnosis**	100	100	98.4
**Type of treatment**	89.2	90.1	91.1
**Experimental stem cell**	98.2	99.1	100
**Primarily funding treatment**	93.7	95.5	93.5
**Beneficiary outside country**	100	100	100
**Beneficiary deceased**	100	100	100

© 2020 Saleh SN et al. *JAMA Network Open.*

eTable 2. Diagnosis Stratified by Type of Treatment Only for Campaigns Primarily Funding Treatment. Values presented as number of campaigns and percent (%) of diagnosis by row for each country.

		Treatment Type for Campaigns Primarily Funding Treatment, n (%)				
Diagnosis by Country		Alternative	Approved – Not Accessible	Experimental – Not Approved	Routine Care	Unspecified
**Acute Illness (n = 28)**	All	2 (7.1)	0 (0.0)	0 (0.0)	25 (89.3)	1 (3.6)
	Canada	0 (0.0)	0 (0.0)	0 (0.0)	4 (80.0)	1 (20.0)
	United Kingdom	1 (11.1)	0 (0.0)	0 (0.0)	8 (88.9)	0 (0.0)
	United States	1 (7.1)	0 (0.0)	0 (0.0)	13 (92.9)	0 (0.0)
**Cancer (n = 614)**	All	144 (23.5)	92 (15.0)	134 (21.8)	224 (36.5)	20 (3.3)
	Canada	49 (34.5)	27 (19.0)	38 (26.8)	23 (16.2)	5 (3.5)
	United Kingdom	66 (24.4)	62 (23.0)	84 (31.1)	47 (17.4)	11 (4.1)
	United States	29 (14.4)	3 (1.5)	12 (5.9)	154 (76.2)	4 (2.0)
**Cardiac (n = 30)**	All	2 (6.7)	4 (13.3)	0 (0.0)	24 (80.0)	0 (0.0)
	Canada	0 (0.0)	0 (0.0)	0 (0.0)	3 (100.0)	0 (0.0)
	United Kingdom	2 (13.3)	4 (26.7)	0 (0.0)	9 (60.0)	0 (0.0)
	United States	0 (0.0)	0 (0.0)	0 (0.0)	12 (100.0)	0 (0.0)
**Neurologic (n = 178)**	All	10 (5.6)	46 (25.8)	75 (42.1)	45 (25.3)	2 (1.1)
	Canada	4 (6.8)	16 (27.1)	31 (52.5)	7 (11.9)	1 (1.7)
	United Kingdom	6 (7.1)	30 (35.7)	36 (42.9)	12 (14.3)	0 (0.0)
	United States	0 (0.0)	0 (0.0)	8 (22.9)	26 (74.3)	1 (2.9)
**Transplant (n = 28)**	All	0 (0.0)	3 (10.7)	1 (3.6)	24 (85.7)	0 (0.0)
	Canada	0 (0.0)	0 (0.0)	1 (33.3)	2 (66.7)	0 (0.0)
	United Kingdom	0 (0.0)	2 (25.0)	0 (0.0)	6 (75.0)	0 (0.0)
	United States	0 (0.0)	1 (5.9)	0 (0.0)	16 (94.1)	0 (0.0)
**Trauma (n = 75)**	All	0 (0.0)	3 (4.0)	8 (10.7)	63 (84.0)	1 (1.3)
	Canada	0 (0.0)	0 (0.0)	2 (22.2)	6 (66.7)	1 (11.1)
	United Kingdom	0 (0.0)	3 (14.3)	3 (14.3)	15 (71.4)	0 (0.0)
	United States	0 (0.0)	0 (0.0)	3 (6.7)	42 (93.3)	0 (0.0)
**Other (n = 126)**	All	16 (12.7)	29 (23.0)	21 (16.7)	49 (38.9)	11 (8.7)
	Canada	3 (10.0)	5 (16.7)	7 (23.3)	10 (33.3)	5 (16.7)
	United Kingdom	7 (9.7)	24 (33.3)	10 (13.9)	30 (41.7)	1 (1.4)
	United States	6 (25.0)	0 (0.0)	4 (16.7)	9 (37.5)	5 (20.8)

© 2020 Saleh SN et al. *JAMA Network Open.*

eReferences

1. Loeb S, Taneja S, Walter D, Zweifach S, Byrne N. Crowdfunding for prostate cancer and breast cancer. BJU Int 2018;122(5):723–5.
2. Snyder J, Caulfield T. Patients' crowdfunding campaigns for alternative cancer treatments. The Lancet Oncology 2019;20(1):28–9.
3. Durand WM, Johnson JR, Eltorai AEM, Daniels AH. Medical Crowdfunding for Patients Undergoing Orthopedic Surgery. Orthopedics 2018;41(1):e58–63.
4. Durand WM, Peters JL, Eltorai AEM, Kalagara S, Osband AJ, Daniels AH. Medical crowdfunding for organ transplantation. Clin Transplant 2018;32(6):e13267.
5. Cohen AJ, Brody H, Patino G, et al. Use of an Online Crowdfunding Platform for Unmet Financial Obligations in Cancer Care. JAMA Intern Med [Internet] 2019 [cited 2019 Oct 20];Available from: https://jamanetwork.com/journals/jamainternalmedicine/fullarticle/2749759
6. Snyder J, Turner L, Crooks VA. Crowdfunding for Unproven Stem Cell–Based Interventions. JAMA 2018;319(18):1935.

© 2020 Saleh SN et al. *JAMA Network Open.*
